# The Use of a CAD/CAM Thermoformed Splints System in Closed Reduction of Condylar Fractures

**DOI:** 10.3390/bioengineering10091023

**Published:** 2023-08-29

**Authors:** Cristina Grippaudo, Antonino Lo Giudice, Gianmarco Saponaro, Mattia Todaro, Alessandro Moro, Antonio D’Addona

**Affiliations:** 1 Dipartimento di Neuroscienze, Organi di Senso e Torace, UOC di Chirurgia Odontostomatologica e Implantologia, Fondazione Policlinico Universitario A. Gemelli, IRCCS, 00168 Rome, Italy; antonio.daddona@unicatt.it; 2 Odontoiatria e Protesi Dentaria, Dipartimento Universitario Testa Collo ed Organi di Senso, Università Cattolica del Sacro Cuore, 00168 Rome, Italy; mattia.todaro@gmail.com (M.T.); alessandro.moro@unicatt.it (A.M.); 3 Department of General Surgery and Surgical-Medical Specialties, Section of Orthodontics, School of Dentistry, University of Catania, 95123 Catania, Italy; antonino.logiudice@unict.it; 4 Dipartimento di Neuroscienze, Organi di Senso e Torace, UOC di Chirurgia Maxillo Facciale, Fondazione Policlinico Universitario A. Gemelli, IRCCS, 00168 Rome, Italy

**Keywords:** CAD/CAM, condylar fractures, IMF, mandibular fractures, maxillomandibular fixation, splints, MMF, thermoformed

## Abstract

(1) Background: Mandibular fractures are very common. Common indications of closed treatment for mandibular fractures are non-displaced or minimally displaced simple fractures in adult compliant patients with good dentition, the absence of occlusal disruption, and fractures in growing children. In closed treatment, the mandible is maintained in centric occlusion with a maxillomandibular fixation (MMF) with orthodontic elastics. Many methods of MMF have been described, often using orthodontic appliances. In recent years, CAD-CAM technology has improved many procedures used in maxillofacial surgery and orthodontics. The device we present is manufactured following a digital workflow, and was designed specifically for MMF. (2) Materials: Two patients with mandibular fractures were treated with an MMF method whose procedure comprised scanning of the dental arches, followed by construction of thermoformed splints on which buttons for the elastics and retention holes are made. The splints were fixed on the dental arches with composite resin at the level of the holes, and were kept in place for the period of healing of the fracture, with the intermaxillary elastics hooked to the buttons. (3) Results: The application time of the splints was very quick. The splints remained stable for the necessary time, without causing particular discomfort to the patients. (4) Conclusions: From our experience, this technique has proved to be reliable and reproducible and could represent a valid tool in the closed treatment of mandibular fractures.

## 1. Introduction

Mandibular fractures account for 10 to 25 percent of all facial injuries, with higher incidence in males (70 to 85%) compared to females (15 to 30%) subjects. Motor vehicle collisions (MVCs) are one of the main causes of mandibular fractures in both sexes, while aggravated assaults are more frequent in males and accidental falls are more frequent in females [1,2,3,4]. Moreover, there are several documented cases of mandibular fractures after sports injuries and gunshot wounds [5,6,7,8].

From the clinical perspective, it is important to establish the treatment strategy for managing clinical fractures, including the decision between the surgical or the conservative approach [1,9,10,11]. The most important factor in deciding whether or not a mandible fracture requires surgical intervention is the status of the occlusion, which can be aberrant in more than 80% of mandibular fractures [12,13]. In this regard, the final goal is to restore the preinjury occlusion, even if it was abnormal. Thus, a preliminary investigation of the type of occlusion present before the trauma is mandatory [14]. It should include a detailed anamnestic analysis to retrieve useful information about bite changes after the trauma; also, historic dental records could be useful to establish any changes occurred after the trauma.

The primary goal in the management of mandibular fractures is restoration of functional occlusion and facial form. Several appropriate techniques are frequently available for the definitive management of a given mandible fracture. In general, mandible fractures are treated either closed (maxillomandibular fixation, splinting, modified diet) or open (plates and screws, interosseous wiring, lag screws). The technique chosen depends on a number of variables, including dental status, fracture characteristics (open, closed, favorable, unfavorable, comminution, bone loss, mechanism of injury, contamination or frank infection, etc.), fracture location, associated injuries, patient mental status, and patient desires. The most important considerations are the dental status, fracture characteristics, and fracture location.

Closed techniques involve the relative fixation of mandibular fragments allowing indirect bone healing, which progresses through granulation, fibrosis, cartilage, and, eventually, bone deposition and remodeling. Closed techniques are best used in cases with favorable, closed fractures and in patients with full dentition. Mandible fractures in children heal quite rapidly and because mixed dentition often precludes the placement of screws in the mandible, closed techniques using some form of maxillomandibular fixation are preferred in many pediatric mandible fractures. Condylar neck fractures have traditionally been managed in a closed fashion with a short course of intermaxillary fixation and soft diet. Despite the nonanatomic reduction in the condylar head, remodeling occurs over a period of months. Maxillomandibular fixation may be supplemented with interosseous wiring of bone fragments, ensuring adequate bone contact and a stable reduction.

Open reduction and internal fixation (ORIF) is the gold standard of treatment for achieving anatomic reduction for a variety of mandibular fractures, including condylar head fractures [15,16] and they have become more common surgical techniques due to the development of more efficient materials. The adoption of these techniques by surgeons has spread, especially in severely fractured, misplaced, and dislocated patients, patients without teeth, patients whose ramus has lost height, and situations in which it is difficult to determine the precise occlusion before trauma. This procedure has been borrowed from orthopedic surgery and has been found to be effective in safely reducing the mandibular fracture. Documented disadvantages of the ORIF are the risk of postoperative infection [17,18,19,20] and the difficulty in restoring the face width and the shape of the dental arch, especially after multi-segmented fractures, generating inconsistencies between dental occlusion and bone fracture reduction. In addition, the ORIF can be extremely difficult; because it is difficult to treat a small area, a fragment is left, resulting in an external scar that may be visible. The procedure is more expensive, requires a more extended stay in the hospital, and increases the chance of wound infection and nerve harm close to arteries such as the internal maxillary artery. The surgical treatment of fractures involves the evaluation of the temporomandibular joint, which may have pathologies or receive damage from the trauma [21].

Non-surgical therapy is indicated in selected cases and may have several advantages over surgical treatment, including lower overall morbidity and risk of ankylosis and avascular necrosis; as for surgical treatment, the occlusal results are more favorable. Maxillomandibular fixation (MMF) is a procedure used in oral and maxillofacial surgery to immobilize or stabilize the upper and lower jaws after bone fracture, with the primary goal of providing stability and promoting proper healing of the jaws or to facilitate specific dental treatments. The MMF is implemented by immobilizing the teeth with a device that allows them to be kept in occlusion through hooks for intermaxillary elastics. The aim is to avoid any force that could displace the fracture stumps during the healing period. MMF represents a more conservative alternative to ORIF and is generally performed using different fixation systems such as Erich arch bars, bone-supported arch bars, direct bonding technique with orthodontic brackets, and passive splinted arch wires [22,23,24,25,26]. However, there are also specific limitations even using this conservative approach, such as difficulty in the maintenance of oral hygiene, periodontal damage, and post-operative discomfort [27]. Detrimental effects on enamel demineralization and gingival irritation may be seen once the arch bar is removed.

Lloyd et al. first reported the use of vacuum-formed splints for the treatment of a mandibular fracture [28].

In 2002, Terai et al. described the use of vacuum-formed, thermoformed plates without elastics for closed treatment of jaw fractures [29].

In 2014, D. V. Trupthi et al. described the use of vacuum-formed IMF splints made from thermoplastic clear foil, designed for both jaws and fixed with glass ionomer cement. They compared it with the arch bar, and the splint showed many advantages in terms of chair side time, periodontal health, patient’s compliance of maintaining oral hygiene, mastication, and speech [30]. These first attempts to use thermoformed splints had the advantage of using tools that were quick and easier to apply. However, they had a coarse appearance and were cemented with glass ionomer cement on all occlusal surfaces. Furthermore, the hooks for the elastics were made of metal, and were applied by drilling the splints in some points which necessarily had to be at least 1.5 mm thick.

In the last 10 years, we have witnessed an innovative process which has seen the increase in the use of thermoformed appliances for orthodontic treatment and for other dental purposes. In addition, there has been an increase in research and production of plastic materials needed in clinical use in terms of elasticity and resistance, even with reduced thicknesses.

Likewise, the use of computer-aided design and computer-aided manufacturing (CAD/CAM) technology has had an impact on oral and maxillofacial surgery [31]. The advantage of the digital workflow is that it speeds up the manufacturing process and enables the generation of devices with greater precision and with an individualized approach [32,33].

In this article, we describe the use and manufacturing of CAD/CAM thermoformed splints used for MMF and closed treatment of mandibular fractures.

The MMF device we propose is the result of research aimed at making MMF operations easier, with an efficient, minimally invasive, and predictable procedure. Our clinical experience with the MMF performed with the application of fixed orthodontic appliances demonstrated the need for expert orthodontic operators, which are not always present alongside the maxillofacial surgeon in our clinical facilities.

Our technique consists of digital dental cast acquisition and creation of two thermoformed splints specially developed for intermaxillary fixation, using a digital workflow and high-performance plastic materials. This allowed us to reduce the thickness of the splints and increase patient comfort.

The aim of this report is to show the clinical usage of this splint, describe the manufacturing process, and present potential advantages, limitations, and recommendations. By way of example, we demonstrate how these new splints were used in two clinical cases with particularities in the occlusion.

## 2. Material and Methods

The thermoformed splint described here was initially produced as a prototype and patented for Italy (N. 202020000005722). The splint requirements were as follows: manufactured with thin plastic materials (0.75 mm), have buttons in plastic material, have buttons distributed along the surfaces and in a number suitable for the need to be able to hook the elastics, be adherent to the dental surfaces and customized, have a good stability while using few points of adhesion with the minimum use of adhesive resins, have a sufficient duration for the retention time without undergoing alterations, allow hygiene, and do not damage the gums.

Patients intended for the use of these splints have the following requirements: presence of a sufficient number of teeth to be able to apply the splints and distribute the elastic traction along the dental arches, have a non-displaced fracture at the level of the jaw, and have the possibility of keeping the mouth open for the time necessary for taking the impressions and the application of splints. The decision to use the scanner for taking impressions was dictated by the need to obtain a high-quality impression in a short time and by introducing the minimum possible thickness into the oral cavity.

The procedure required a first intervention to take the scans of the dental arches followed by the fabrication of the splints. Scans were performed with the 3Shape Trios 3 scanner (3 Shape, Copenhagen, Denmark). The small size of the instrument made it possible to perform the procedure with a discomfort that was bearable by the patients. Scan time was within the norm, approximately 5 min per arch.

From the STL files of the scans, the models of the arches were printed. Thermoformed splints were prepared using a sheet of polyvinyl chloride 0.75 mm thick Erkodur (ERKODENT^®^, Siemensstraβe 3, D-72285, Pfalzgrafenweiler, Germany).

In the laboratory, the splints were machined to have some thermoformed buttons and some holes for retention of the composite. The buttons were manufactured using the special pliers of the ThermalOrth^®^ system (Orthodontic H.D. snc, Bologna, Italy). The position of the buttons was established on the basis of the need to have a retention for the intermaxillary elastics in a lateral and anterior position.

The number and position of the buttons were established on an individual basis, considering the number and shape of the teeth. These must be sufficient to guarantee the good hold of the elastics and the maintenance of the occlusion relationship.

For the retention of the thermoformed splints, holes were made using a bur (HM41 023, Hager & Meisinger GmbH, Hansemannstraße 10, 41468 Neuss, Germany) at the vestibular surface of the dental crowns; at least two were made in the premolar and molar areas and one on the incisors.

**Figure 1:**
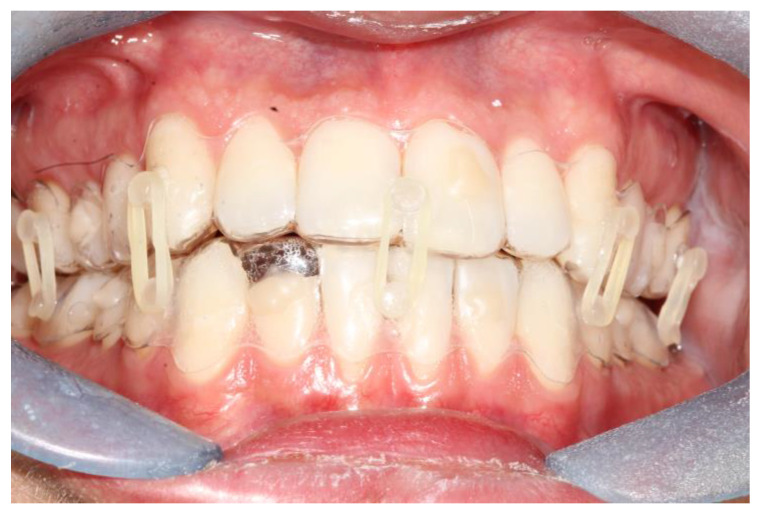
Intraoral view of patient 2. It is possible to see the coronal fracture of element 4.2; the fitting of the splints is adequate and so is the fixation.

**Figure 2:**
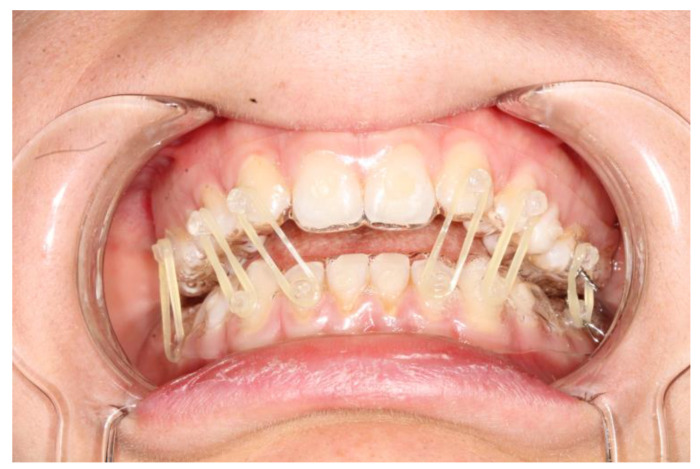
Intraoral view of patient 1; the fitting of the splints is adequate and so is the fixation.

The splints were bonded to the teeth with a restorative flowable composite (Filtek Supreme Flowable, 3M, St. Paul, MN, USA), which was applied through the holes designed on the splint (Figure 1 and Figure 2).

The time taken to place the splints was very short, limited to the polymerization time of the composite. The VALO^®^ Ortho Cordless Curing Light (Opal Orthodontics, 505 West 10200 South, South Jordan, UT 84095, USA) was used to cure the composite.

Orthodontic elastics were used to obtain the intermaxillary fixation.

To demonstrate the possibilities of clinical use, we show two cases of young patients, affected by non-displaced mandibular fractures, in which it would have been difficult to use a MMF with brackets and orthodontic wire.

They were adult and compliant, without avulsed teeth due to the trauma, and without other comorbidities or fractures.

**Figure 3:**
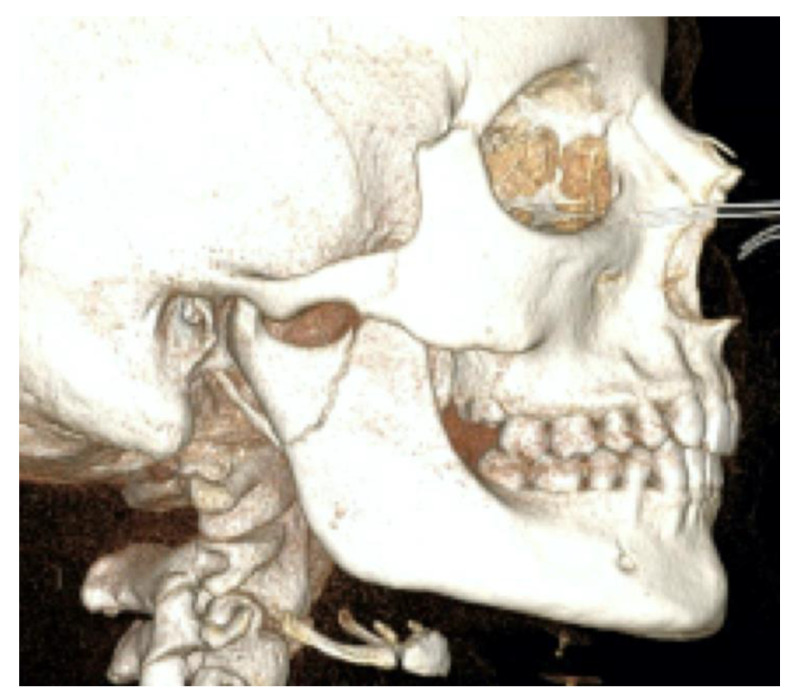
Patient 1 with a right sub-condylar non-displaced fracture, with sound permanent dentition and without comorbidities.

The first patient was a woman with a right sub-condylar non-displaced fracture, with sound permanent dentition, and without comorbidities (Figure 3). She had already received orthodontic treatment for agenesis of the upper lateral incisors, with space closure by means of mesialization of the upper teeth. She had a molar class II, the upper canines in the place of lateral incisors, and a deep bite. In this case, the application of a fixed orthodontic appliance would have implied the need to be absolutely passive in order not to compromise the result of the orthodontic therapy. Furthermore, the anterior deep bite would have been an impediment for the correct positioning of the brackets on the lower incisors.

**Figure 4:**
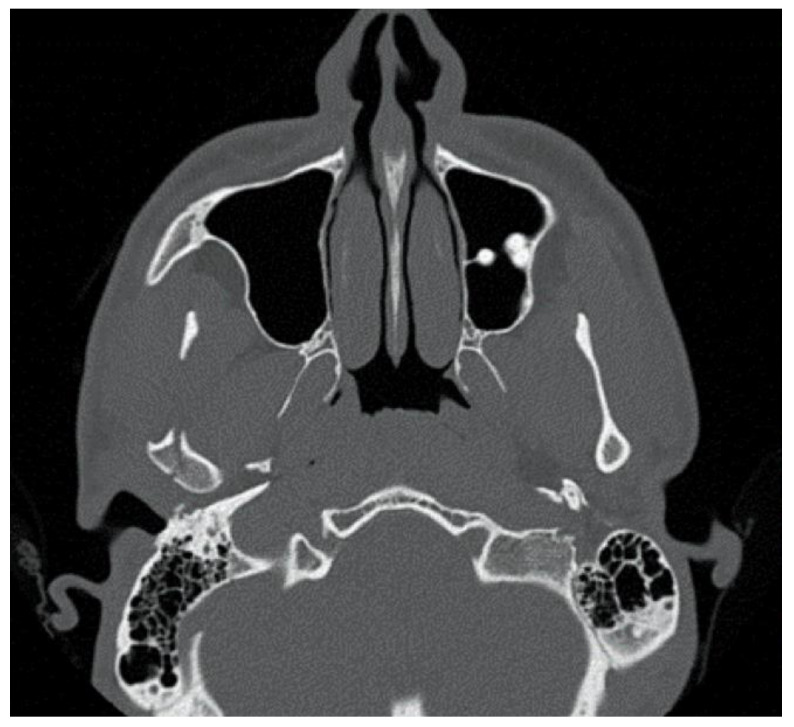
Patient 2, male, aged 20, with a right condylar diacapitular fracture.

The second patient was a male, aged 20, with a right condylar diacapitular fracture (Figure 4). The patient also presented a coronal fracture of the element 43 due to the trauma. The other teeth were sound, but the 37 was unerupted. He presented a slight class III malocclusion and a posterior open bite on the left. In this case, there was the need to not exert force on the fractured tooth, and to balance the traction points of the rubber bands despite the absence of the 37, avoiding extrusive forces on the 27.

The two patients were treated by closed reduction between March and June 2022.

The period of intermaxillary fixation was 20 days, during which patients had to wear elastics all the time to keep the mouth closed, and to replace these once a day.

After the application of the retention devices, patients were instructed to follow a liquid diet. They were advised to maintain oral hygiene by gently brushing the splints with a soft toothbrush and rinsing with 0.12% chlorhexidine mouthwash once a day.

During the retention time, they were visited after one week to check the ongoing therapy.

After the retention period, the devices were removed and a cleaning procedure was conducted to remove the residual composite from the teeth. The resin was removed using a 12-blade tungsten carbide bur (C22AGK, Edenta, Schaanwald, Liechtenstein) driven on a 20,000 rpm low-speed handpiece without water cooling. This procedure was followed by finishing the residual resin with sandpaper discs (Sof-Lex, 3M, St. Paul, MN, USA) from medium to ultrafine, driven on a 10,000 low-speed handpiece, and polishing with pumice prophylaxis paste (SuperPolish, Kerr, Bioggio, Switzerland) and water.

At the end of the IMF period, a control CT (computed tomography) scan was obtained for every patient.

After verification of the CT scan, the patients were instructed to continue the liquid diet for another 2 weeks and to gradually increase the jaw opening.

## 3. Results

All patients recovered without complications.

All patients were able to follow a liquid diet during the IMF period.

**Figure 5:**
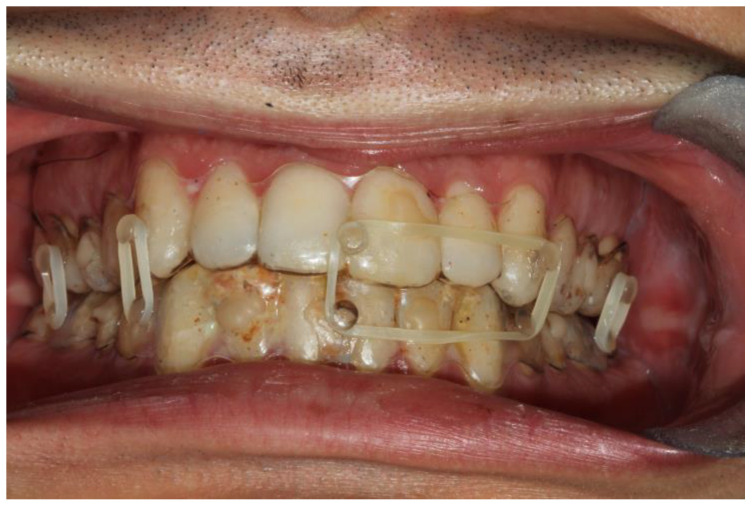
Intraoral view of patient 2 during the treatment; is it possible to note the slight problem with oral hygiene, which is nonetheless completely acceptable.

**Figure 6:**
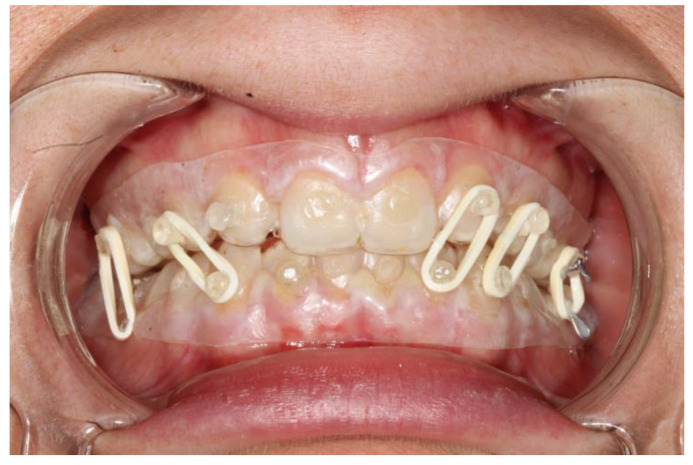
Intraoral view of patient 1 during the treatment; is it possible to note the slight problem with oral hygiene, which is nonetheless completely acceptable.

The fixation strength of the intermaxillary fixation was considered adequate in both cases (Figure 5 and Figure 6).

Occlusion and periodontal tissue were within normal clinical ranges after the treatment (Figure 6).

**Figure 7:**
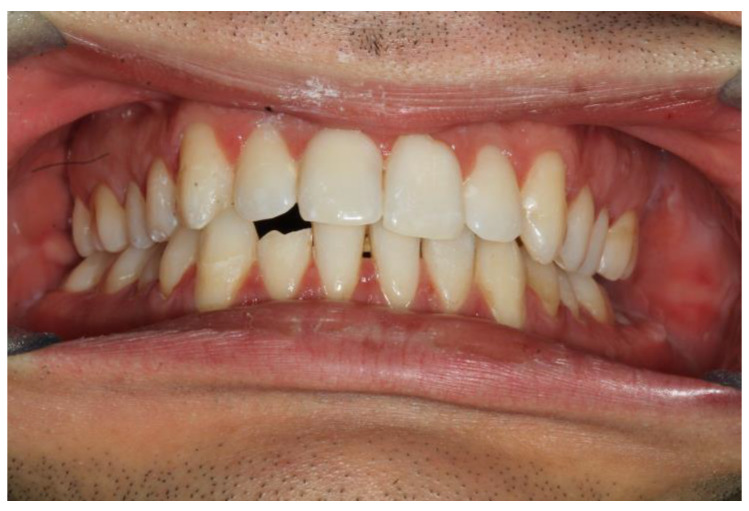
Patient 2 after splint removal and professional hygiene; is it possible to note the good restoration of the occlusion.

Only in the first patient, where the splint margin covered the attached gingiva, was some periodontal inflammation noted (Figure 7).

The major problem reported by the male patient was weight loss and asthenia due to the liquid diet.

## 4. Discussion

The mandibular condyle is a common site of fracture, usually because of trauma. The best way to treat such fractures has been the subject of lengthy debate and discussion between oral and maxillofacial surgeons for many years. Treatment of such fractures can be either by surgical open reduction and fixation, or by a much simpler closed method, in which the patient is given rigid intermaxillary fixation or, more commonly, intermaxillary elastic traction. An important potential complication of the open approach is damage to the facial nerve. Other techniques, including endoscopic approaches, are used in some centers. Condylar fractures commonly occur in conjunction with at least one other fracture elsewhere in the mandible, which usually require open reduction and fixation; this may influence the choice of treatment of the condyle.

A major concept in the treatment of facial fractures is that dental occlusion can be used as a guide for fracture reduction and as a therapeutic tool to correct and maintain jaw position as the bone heals.

Intracapsular fractures of the mandibular condyle are classified as type A—fractures through the medial condylar pole; type B—fractures through the lateral condylar pole with loss of vertical height of the mandibular ramus; or type M—multiple fragments, comminuted fractures. The majority of mandibular condyle fractures involve the condylar neck, with few reports of intracapsular fractures. Sagittal or vertical fractures of the mandibular condyle and chip fractures of the medial part of the condylar head are rarely found by conventional radiography and are more commonly detected by computed tomography (CT) scan [10].

For moderately displaced condylar fractures, closed treatment with rigid or elastic maxillomandibular fixation is still frequently selected. The reasons for this may be the difficult surgical access to the condylar area and the frequently difficult repositioning of the proximal fragment. Open reduction and internal fixation of condylar fractures may be indicated for bilateral injuries or considerably displaced condylar fractures, but closed treatment and intermaxillary fixation (IMF) may be indicated in cases where condylar displacement is minimal and the height of the ramus is almost normal.

Functional therapy (closed treatment) is adopted most frequently since it permits early mobilization and adequate functional stimulation of condylar growth (in growing subjects) and bone remodeling (in all subjects). It is indicated in almost all condylar fractures that occur in childhood, and in intracapsular and extracapsular fractures that do not include serious condylar dislocation in adults. In contrast, surgical treatment is indicated primarily for adults with displaced fractures or with dislocation of the condylar head.

Currently, intermaxillary fixation can be achieved effectively with different methods; however, most of these present clinical issues and a high level of discomfort for the patient. In this regard, intermaxillary fixation with fixed orthodontic appliances (bonded brackets) has been widely used for the clinical management of maxilla-mandibular fractures. However, this technique has two major disadvantages. The first limitation is related to the time needed to bond the brackets, during which the patient must keep his mouth dry and kept open by a cheek retractor. This procedure generates discomfort to the patient and is not exempt from bonding failure due to the difficulty in controlling the contamination of the saliva. The second limitation is linked to the need to stabilize the position of the teeth with a rectangular and rigid orthodontic wire, which is bent to adapt passively to the position of the teeth, without stressing them with orthodontic forces. This procedure requires time in bending the wire, as well as manual skills, and its application should be discouraged in the absence of adequate manual and clinical experience.

To overcome these drawbacks, other methods have been proposed, including the use of splints to be bonded or fixed to the teeth, some of which are thermoformed [12,13,14]. The main advantages of using splints for intermaxillary fixation are (1) the possibility to minimize the risk of stressing individual teeth with orthodontic forces and (2) the greater efficiency of appliance placement and maxilla-mandibular fixation [34].

Compared to the previous versions of splints proposed in literature for the management of mandibular fracture, the present method has the novelty of using a digital workflow and making the anchor buttons directly on the splints. The fixing method is also original and simple to perform.

The usage of a digital workflow in the manufacturing process of splints introduces specific enhancements in the clinical management of intermaxillary fixation. The registration with an intra-oral scanner is notably more comfortable compared to analogic impression and, with an appropriate scan protocol, can be performed without significant mouth opening [35]. This is extremely important in post-traumatic patients. Secondly, .stl files can be stored and are immediately available in case it is necessary to reproduce the appliance, for example, after accidental breaking. Thirdly, the digital scan can be visualized by both orthodontist and maxillofacial surgeons using the same digital platform, allowing better communication and cooperation (for example, in the choice of where to put buttons) [36].

Concerning the design of the splints proposed in the present manuscript, we opted to select specific areas to generate holes that serve to release the composite used to fix the splint. This procedure provides great stability to the appliances counteracting the tendency to slip from occlusal surfaces caused by the intermaxillary elastics. At the same time, anchor buttons were designed on the thermoformed splint using specific pliers (ThermalOrth^®^ system—Orthodontic H.D. snc, Bologna, Italy), avoiding the necessity to bond anchor buttons or eyelets upon dentition.

Some considerations should be addressed concerning the possibility to replace thermoformed splints with direct 3D-printed splints, according to our protocol. Firstly, when using direct-printed splints, it is necessary to set the appropriate printing parameters, including the appliance offset (which are different among 3D-printing devices and resins), in order to find the best compromise between appliance fitting and retention [37,38]. Furthermore, to digitally design splints, specific equipment (and software) or consolidated experience in digital design process are required, limiting this option to skilled clinicians or when prescribing the appliance to the lab technician. Presumably, greater complexity of the procedure and higher costs would not produce significant clinical benefits.

It should be underlined that the thickness of the splints proposed in the present study is 0.75 mm, that is, the same thickness as conventionally used in orthodontics for clear aligners [39,40,41]. In MMF, the thickness of the material used to cover the occlusal plane must be minimal in order not to alter the relationship between the jaws and risk changing the position of the fracture stumps.

In the light of the abovementioned considerations, the protocol proposed, i.e., digital intra-oral acquisitions, model printing, and thermoforming splints represent an excellent and versatile solution applicable in clinical settings for managing intermaxillary fixation.

In addition, it should be borne in mind that patients with a condyle fracture have pain when they keep their mouth open, due to edema following the contusion. Therefore, it is necessary to limit the amount and time the mouth is opened, in order not to risk displacing the fractured bone segments. With the method we propose, it is possible to create a customized device that is easy to use and allows good stability of the intermaxillary fixation for the clinician, and is also very comfortable for the patient. The digital workflow allows the construction of the device in the laboratory, reducing chair time [42].

The surface of the appliance does not have any roughness that could scratch or irritate the mucous membranes of the cheeks. The patient can brush the oral hygiene appliance without risk of damaging it. Naturally, hygiene cannot be optimal, as always in the case of intermaxillary fixation, and the use of a mouthwash must be combined with brushing. In our patients, after 20 days of using the equipment and feeding on a liquid diet, we observed the presence of moderate gingivitis, easily treatable with improved oral hygiene procedures.

A limitation of the system used can be identified in the button manufacturing procedure, which could be made faster by gluing pre-formed buttons on the splints. The production of new adhesive materials that allow resin or metal buttons to adhere to the surface of the splints can further improve the production process, albeit with a minimal increase in costs.

## 5. Conclusions

The use of thermoformed splints equipped with buttons for the application of intermaxillary elastics for the containment of simple fractures of the mandibular condyle has been proposed.

The advantages of the device are as follows:

- Reduced time in the chair and, above all less, time in which it is necessary for the patient to be open-mouthed.
- Custom-made appliance, which adapts perfectly to all occlusal situations and does not exert unwanted forces on the dental elements.
- Good retention and good clinical result in remaining effective and efficient for as long as necessary.
- Good tolerance on the part of the patient and less aesthetic impact than fixed appliances with brackets.

In conclusion, this technique provides reliable and reproducible outcomes, and represents a useful tool in the closed treatment of mandibular fractures.

## Data Availability

Data available on request due to restrictions of privacy or ethical. The data presented in this study are available on request from the corresponding author.
